# Application of Fast Non-Local Means Algorithm for Noise Reduction Using Separable Color Channels in Light Microscopy Images

**DOI:** 10.3390/ijerph18062903

**Published:** 2021-03-12

**Authors:** Seong-Hyeon Kang, Ji-Youn Kim

**Affiliations:** 1 Department of Radiological Science, College of Health Science, Gachon University, 191, Hambakmoero, Yeonsu-gu, Incheon 21936, Korea; tjdgus7345@gachon.ac.kr; 2 Department of Dental Hygiene, College of Health Science, Gachon University, 191, Hambakmoero, Yeonsu-gu, Incheon 21936, Korea

**Keywords:** fast non-local means (FNLM) using separable color, various control parameters of FNLM, noise reduction, light microscopy (LM) image, quantitative evaluation of image characteristics

## Abstract

The purpose of this study is to evaluate the various control parameters of a modeled fast non-local means (FNLM) noise reduction algorithm which can separate color channels in light microscopy (LM) images. To achieve this objective, the tendency of image characteristics with changes in parameters, such as smoothing factors and kernel and search window sizes for the FNLM algorithm, was analyzed. To quantitatively assess image characteristics, the coefficient of variation (COV), blind/referenceless image spatial quality evaluator (BRISQUE), and natural image quality evaluator (NIQE) were employed. When high smoothing factors and large search window sizes were applied, excellent COV and unsatisfactory BRISQUE and NIQE results were obtained. In addition, all three evaluation parameters improved as the kernel size increased. However, the kernel and search window sizes of the FNLM algorithm were shown to be dependent on the image processing time (time resolution). In conclusion, this work has demonstrated that the FNLM algorithm can effectively reduce noise in LM images, and parameter optimization is important to achieve the algorithm’s appropriate application.

## 1. Introduction

Light microscopy (LM) is employed to capture magnified high-resolution images of objects invisible to unaided sight. In addition, LM hardware and software technologies have rapidly advanced for application in various fields. In particular, LM images have considerably contributed to the comprehension of human mechanisms by providing functional and structural information on specimens, such as cells and tissues, in biomedical research [1,2]. Although the technologies and application methods for LM images have been actively developed, the analysis of several colorless and transparent specimens is difficult because the structures are indistinct and the background contrast is insufficient. To clearly examine such specimens, various staining techniques have been developed to obtain color LM images [3,4] in which the contrast between the specimen and background is high and the separation among various tissues is distinct [5,6].

In capturing LM images, hardware defects, such as focal length and lens misalignment, and the physical properties of light, such as scattering and quantum discreteness, typically generate noise [7]. Noise leads to the inaccurate analysis of observed specimens because the structure and component information of images are distorted [8]. To resolve this, various algorithms to reduce noise have been developed [9,10,11]. However, the application of conventional noise reduction algorithms causes a blurring effect that negatively affects the sharpness and resolution of LM images [12,13]. In particular, the blurring effect on LM images has been pre-generated owing to the refraction and diffraction of the light source. Moreover, the application of noise reduction algorithms can aggravate the effect of this undesirable phenomenon [14,15,16]. Accordingly, noise reduction algorithms using the latest technologies, such as total variation, wavelet transform, deep-learning, and non-local means (NLM), have been proposed to minimize the blurring effect and selectively remove only noise [17,18,19,20]. Among the noise reduction algorithms mentioned above, the NLM algorithm is a known method that can smooth the pixel value inside the edge structure and maintain the high-frequency signal by calculating the intensity and distance of neighboring pixels [21,22,23].

However, the practical implementation of the NLM algorithm to various fields, including LM systems, is difficult, because the calculation of distance weights is excessively time-consuming [24,25]. In particular, the application to color LM images is more exigent due to the increase in the amount of data that has to be acquired from at least three channels (e.g., red, green, and blue (RGB) channels) [26,27,28]. To overcome the excessive time consumption of the NLM algorithm, the fast non-local means (FNLM) algorithm with the vectorization of a two-dimensional distance-weighted equation to one that is one-dimensional is proposed. In contrast to the NLM algorithm, the operational amount of the FNLM algorithm, to which an improved weighting equation is applied, is determined independently of the local patch size. The improved operational amount provides an efficient and fast image processing time. In particular, the advantage for the processing time of the FNLM algorithm can be applied effectively to color LM images with a high resolution and multiple channels. In addition, the modeled FNLM algorithm has various parameters, such as smoothing factor, search window, and kernel size [29,30]. These parameters should be appropriately set for the situation and purpose of the image analysis, because they considerably influence the image resolution and characteristics.

Thus, the purpose of this study was to analyze the tendency of image characteristics with changes in the various parameters for the effective application of the proposed FNLM algorithm in color LM images.

## 2. Materials and Methods

### 2.1. Acquisition of Light Microscopy Image

The LM images of immunohistochemically stained gingival sections showing the distribution of the anti-Ki-67-labeled cells were obtained using a digital microscope (DM500; Leica Microsystem, Heerbrugg, Switzerland), Leica ICC50 E camera (Leica Microsystem, Heerbrugg, Switzerland), and the Leica LAS EZ software (Leica Microsystem, Heerbrugg, Switzerland). Ki-67, as a proliferation marker, labeled proliferating nuclei in a brown color. A counterstain was performed with hematoxylin to make the immunohistochemically stained tissue structure easily visible. Hematoxylin gives clear nuclear staining. The acquired image showed brown (for immunopositive) and blue (for immunonegative) nuclei.

### 2.2. Fast Non-Local Means Algorithm Modeling

The conventional local filters exhibited an excellent performance in noise reduction. However, a blurring effect occurred because only the calculated pixel values inside the set kernel were reflected. To solve this problem, A. Buades et al. proposed the use of the NLM algorithm, which can calculate the intensity difference and relative distance between the target and neighboring pixels. The operation of the proposed image processing algorithm can eliminate the noise and maintain high-frequency signals, such as those found in the edge regions, as follows:

(1) $$NL\left[f\right]\left(m\right)=\;\sum_{n=I}w\left(m,n\right)f\left(n\right),$$

where f denotes the noisy image; m and n are the pixel values in the noisy image; and w(m,n) is the distance weight that depends on the similarity of pixels m and n, satisfying the condition 0≤w(m,n)≤ 1. The similitude between m and n is related to the similarity of the intensity of gray level vectors, $v\left(k_{m}\right)$ and $v\left(k_{n}\right)$, where $k_{i}$ denotes the square-shaped kernel centered on pixel i. In addition, the distance weight, w(m,n), in Equation (1) is as follows:

(2) $$\omega \left(m,n\right)=\frac{1}{Z\left(m\right)}e^{-\frac{\|v\left(k_{m}\right)-v\left(k_{n}\right){\|}_{2,a}^{2}}{d^{2}}},$$

where Z(m) is the normalizing constant, and

(3) $$Z\left(m\right)=\;\sum_{n}e^{-\frac{\|v\left(k_{m}\right)-v\left(k_{n}\right){\|}_{2,a}^{2}}{d^{2}}},$$

where $\|v\left(k_{m}\right)-v\left(k_{n}\right){\|}_{2,a}^{2}$ denotes the weighted Euclidean distance, in which the Gaussian kernel with a standard deviation of a is applied and d is the smoothing parameter that controls the degree of filtering. However, the NLM algorithm inefficiently calculates the distance weight; thus, we have attempted to improve this estimation process. The distance weight can be interpreted as follows:

(4) $$\omega \left(m,n\right)=\frac{1}{Z\left(m\right)}\sum_{n}e^{-\frac{G_{a}\left(\lambda \right)\|f\left(m+\lambda \right)-f\left(n+\lambda \right){\|}_{2}^{2}}{d^{2}}},$$

where $G_{a}\left(\lambda \right)$ denotes the Gaussian distribution size, $a^{2}$, for the number of pixels, λ, of the set kernel, and $\|f\left(m+\lambda \right)-f\left(n+\lambda \right){\|}_{2}^{2}$ is the intensity difference between pixels m and n based on the Euclidean distance. The FNLM algorithm was modeled by replacing the distance weight, which was calculated in the two-dimensional equation using a one-dimensional equation. The equation of the modified distance weight is as follows:

(5) $$\overset{ˇ}{\omega }\left(m,n\right)=\frac{1}{Z\left(m\right)}S_{i}\left(f\left(m+P\right)-f\left(n-P\right)\right),$$

(6) $$S_{i}\left(p\right)=\;\sum_{\tau =0}^{p}e^{-\frac{\|f\left(\tau \right)-f\left(\tau +\overset{ˇ}{\lambda }\right){\|}_{2}^{2}}{d^{2}}},$$

where *P* denotes the local patch size when the image is vectorized in one dimension and $\overset{ˇ}{\lambda }$ and p are defined as n−m and $m+\;\overset{ˇ}{\lambda }$, respectively. If the required operational quantity of the NLM algorithm is $O{\left(P\right)}^{dimension}$, then the FNLM algorithm with the proposed equation is determined as $O(2^{dimension})$. Modeling the FNLM algorithm based on the modified distance weight can improve the time resolution of image processing to reduce noise.

### 2.3. Application of Fast Non-Local Means Algorithm

The modeled FNLM algorithm has various parameters. Among these, the smoothing factor is set to determine the filtering degree. This filtering degree is mutually exclusive with the sharpness and resolution of images; hence, an appropriate smoothing factor value should be used with the FNLM algorithm. In addition, the search window and kernel sizes are controlled to yield distance weights based on the Euclidean function. These parameters determine the computational complexity, which has a major role in image processing. 

**Figure 1:**
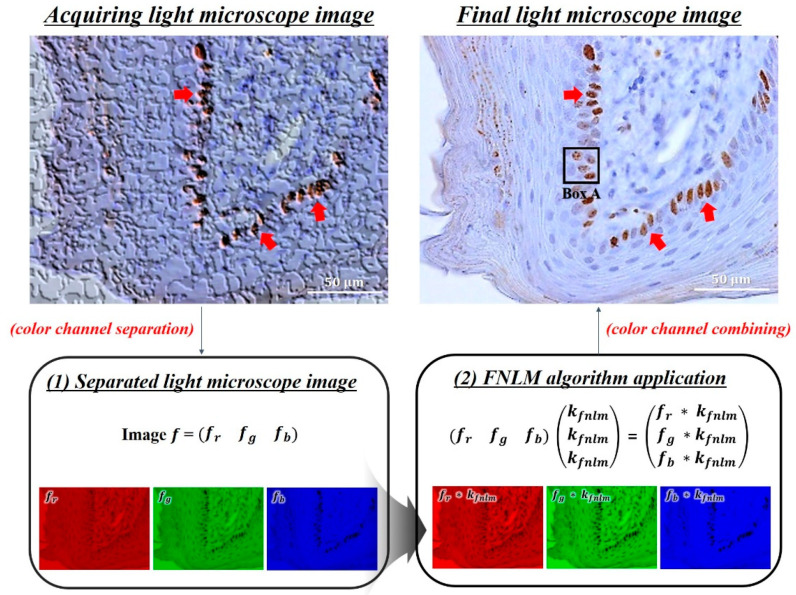
Simplified flowchart of applying the fast non-local means (FNLM) algorithm (at 400× magnification) to light microscopy (LM) images (scale bar = 50 μm).

To analyze the image characteristics according to the change in the FNLM algorithm parameters, an experiment on the smoothing factor, which has the most considerable effect on noise reduction, was preferentially performed by the authors. The experiment was performed using various smoothing factor values (i.e., 0.01, 0.0125, 0.015, 0.02, 0.025, 0.03, 0.035, 0.04, 0.045, and 0.05) in the FNLM algorithm for the color LM image. The search window and kernel sizes were set as 7 × 7 and 21 × 21, respectively. Thereafter, an experiment on the search window and kernel sizes was conducted. For this, the kernel sizes were 3 × 3, 5 × 5, 7 × 7, 9 × 9, and 11 × 11, and the search window sizes were 11 × 11, 21 × 21, 31 × 31, and 41 × 41; the optimized smoothing factor values derived through the preferential experiments. To employ the FNLM algorithm, the RGB channels of the color LM image were separated. Then, using the same parameters, the algorithm was applied to each of the separated channels. Finally, the filtered color LM image was acquired by combining each of the three channels to which the FNLM algorithm is applied. Figure 1 shows the simplified flowchart of applying the FNLM algorithm to the color LM image.

### 2.4. Quantitative Evaluation

The coefficient of variation (*COV*) was measured to evaluate the denoising performance of the FNLM algorithm, as follows:

(7) $$COV=\;\frac{\sigma_{r}}{\mu_{r}},$$

where $\sigma_{r}$ and $\mu_{r}$ are the standard deviation and mean in the set region of interest, respectively.

In addition, the blind/referenceless image spatial quality evaluator (BRISQUE) and natural image quality evaluator (NIQE), which are blind image quality assessment factors, were used to evaluate the degree of image restoration. The BRISQUE learned mapping with the measured features based on the converted luminance using the mean subtracted contrast normalized (MSCN) coefficient. The MSCN coefficient is as follows [31]:

(8) $$\hat{f}\left(m,n\right)=\;\frac{f\left(m,n\right)-\;\hat{\mu }\left(m,n\right)}{\hat{\sigma }\left(m,n\right)+C},\;$$

(9) $$\hat{\mu }\left(m,n\right)=\;\sum_{k=-K}^{K}\sum_{l=-L}^{L}\varphi_{k,l}f_{k,l}\left(m,n\right),$$

(10) $$\hat{\sigma }\left(m,n\right)=\;\sqrt{\sum_{k=-K}^{K}\sum_{l=-L}^{L}\varphi_{k,l}{\left(f_{k,l}\left(m,n\right)-\hat{\mu }\left(m,n\right)\right)}^{2}},$$

where C = 1 is a constant for preventing the denominator from becoming 0 and $\hat{\mu }\left(m,n\right)$ and $\hat{\sigma }\left(m,n\right)$ are the local mean field and local variance field, respectively, obtained by applying the Gaussian filter, $\varphi_{k,l}$. Moreover, the empirical distribution of the pairwise products of MSCN coefficients was obtained along the four directions and used to model the statistical relationship between two neighboring pixels as follows:

(11) $$H\left(m,n\right)=\;\hat{f}\left(m,n\right)\hat{f}\left(m,n+1\right),$$

(12) $$V\left(m,n\right)=\;\hat{f}\left(m,n\right)\hat{f}\left(m+1,n\right),$$

(13) $$D_{1}\left(m,n\right)=\;\hat{f}\left(m,n\right)\hat{f}\left(m+1,n+1\right),\;$$

(14) $$D_{2}\left(m,n\right)=\;\hat{f}\left(m,n\right)\hat{f}\left(m+1,n-1\right).$$

The features of the acquired paired products were extracted based on the asymmetric generalized Gaussian distribution model. The acquired features were learned using a support vector regressor to measure the image quality score. 

The NIQE shows the image quality as a score by analyzing the probability density function of the multivariate Gaussian distribution for the derived features with selected multiple patches. To model the NIQE, the MSCN coefficients of the image were obtained and divided into equally sized patches. Then, the features were derived using the same method as the BRISQUE. The derived features were employed to calculate the mean vector and covariance matrix. Finally, the similarity was measured based on the calculated average vector and covariance matrix, as well as the MSCN coefficient of the original image, as follows [32]:

(15) $$D\left(v_{1},v_{2},\;\emptyset_{1},\emptyset_{2}\right)=\;\sqrt{{\left(v_{1}-v_{2}\right)}^{T}{\left(\frac{\emptyset_{1}+\emptyset_{2}}{2}\right)}^{-1}\left(v_{1}-v_{2}\right)},$$

where $v_{1}$, $v_{2}$ and $\emptyset_{1}$, $\emptyset_{2}$ are the mean vectors and covariance matrices of the original image, respectively. Smaller mean values of the NIQE and BRISQUE can improve image characteristics.

## 3. Results

### 3.1. Smoothing Factor Experiment

**Figure 2:**
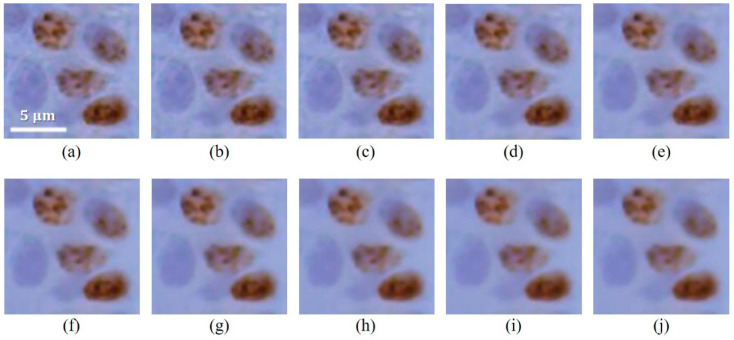
Color LM images filtered using FNLM algorithm (scale bar = 5 μm) with various smoothing factors: (**a**) 0.01, (**b**) 0.0125, (**c**) 0.015, (**d**) 0.02, (**e**) 0.025, (**f**) 0.03, (**g**) 0.035, (**h**) 0.04, (**i**) 0.045, and (**j**) 0.05.

The immunohistochemically stained gingival sections were imaged at on-microscope magnifications of 400× conditions. Subsequently, an experiment was performed to analyze the effect of the FNLM algorithm with various smoothing factors on the color LM image characteristics. Figure 2 presents the magnified regions (corresponding to Box A in Figure 1) of the filtered color LM images using the FNLM algorithm with different smoothing factors.

**Figure 3:**
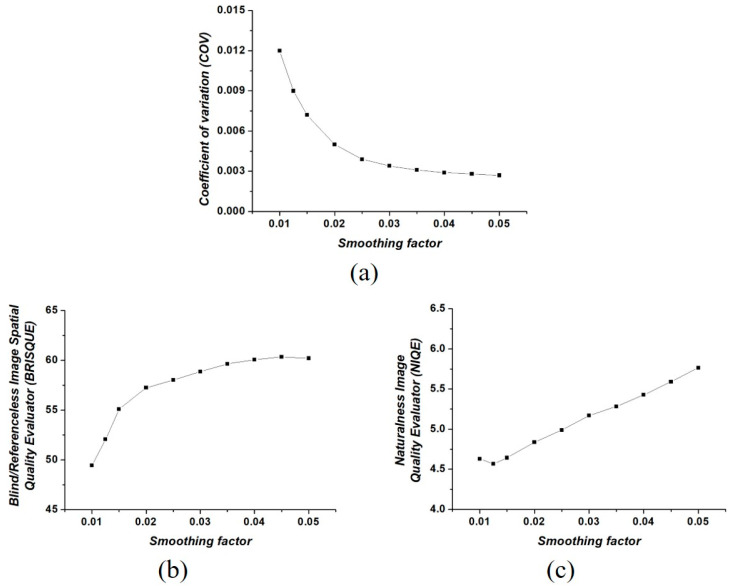
(**a**) Coefficient of variation (COV), (**b**) blind/referenceless image spatial quality evaluator (BRISQUE), and (**c**) natural image quality evaluator (NIQE) results of filtered color LM images using the FNLM algorithm with various smoothing factors.

Figure 3 shows the COV, BRISQUE, and NIQE results of filtered color LM images using the FNLM algorithm with various smoothing factors. All three evaluation parameters exhibited a consistent trend as the smoothing factor increased. 

### 3.2. Search Window and Kernel Size Experiment

**Figure 4:**
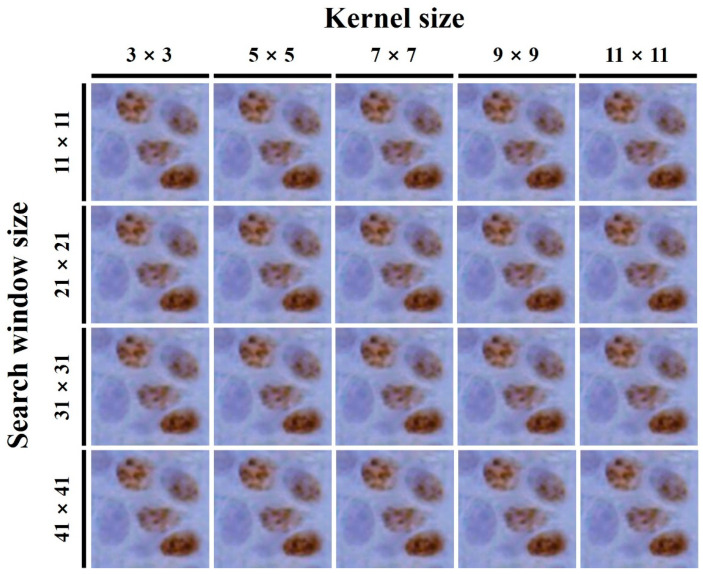
Color LM images filtered using the FNLM algorithm with various kernel and search window sizes.

**Figure 5:**
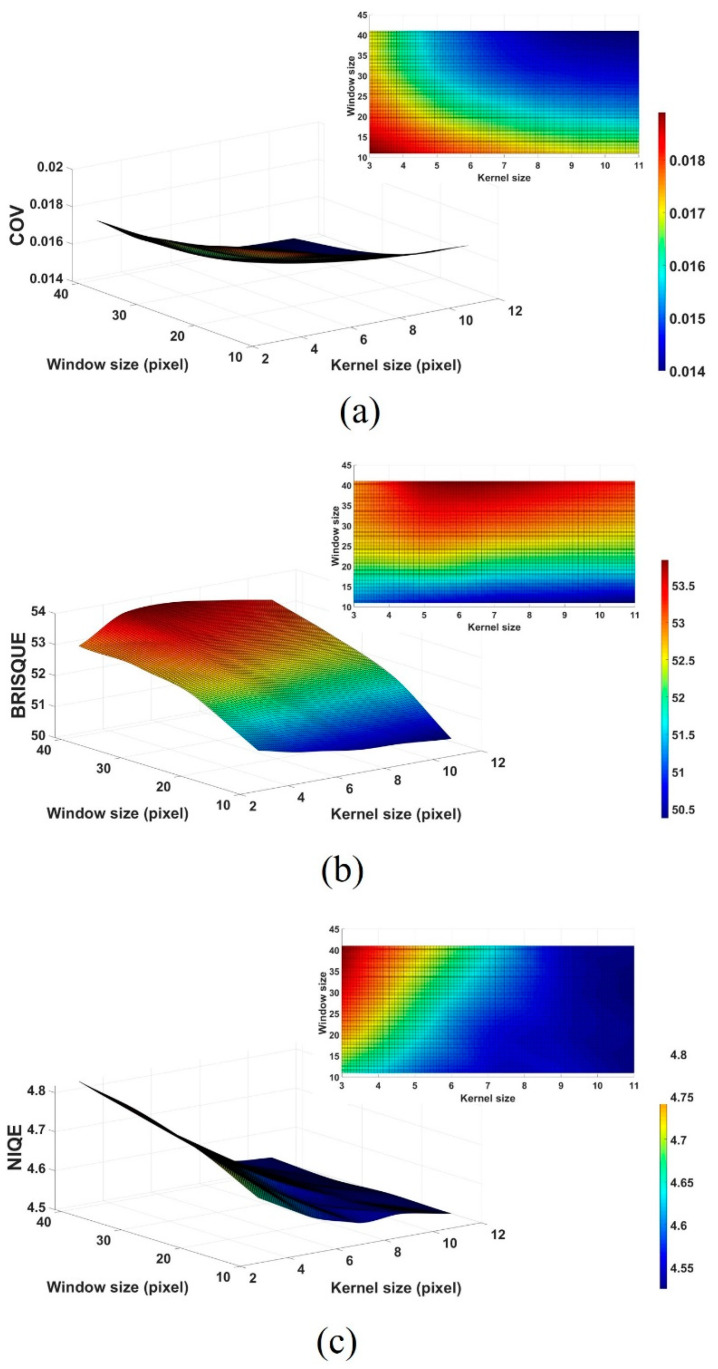
(**a**) COV, (**b**) BRISQUE, and (**c**) NIQE results of filtered color LM images processed by the FNLM algorithm with various search window and kernel sizes.

An experiment was conducted to analyze the effect of the FNLM algorithm with various search window and kernel sizes on image characteristics. In analyzing the COV, BRISQUE, and NIQE results according to the smoothing factor, no significant differences between the 0.01 and 0.0125 values were observed. Thus, the image using a 0.0125 smoothing factor, which showed slightly better results in NIQE, was used as the reference to confirm the change in image quality according to the search window and kernel sizes. Figure 4 presents the magnified regions and Figure 5 shows the COV, BRISQUE, and NIQE results of the filtered color LM images using the FNLM algorithm with various search window and kernel sizes.

## 4. Discussion

Noise is typically generated when LM is employed to capture images. Moreover, in the biomedical field where cells and tissues with complex structures and functions are analyzed, the presence of noise is fatal [33,34]. Although various conventional algorithms have been developed to reduce and solve noise problems, these processes have deteriorated the sharpness and resolution of LM images [35,36,37].

The NLM algorithm estimates new pixel values by assigning distance weights to neighborhood pixel values using an evaluation based on similarity; hence, it can overcome the disadvantages of conventional noise reduction algorithms. However, estimating the distance weights using the NLM algorithm requires substantial amounts of calculation, which consume excessive computational time [38,39,40]. This problem is further aggravated when processing high-resolution LM images, because such images contain large amounts of data. In particular, the color LM image of a stained specimen has more than three times the amount of data of a single-channel LM image. Thus, the use of the modified method in the NLM algorithm to process color LM images is necessary.

Accordingly, the FNLM algorithm was modeled by the vectorization of the one-dimensional equation for distance weight and applied to color LM images by separating each channel. In addition, various evaluation factors were measured to confirm the effect of the FNLM algorithm parameters on color LM images.

An experiment on the smoothing factor, which has the foremost influence on denoising efficiency among the parameters, was preferentially conducted. As shown in Figure 3a, the COV exponentially decreases as the smoothing factor increases because the degree of smoothing also increases. In particular, the COV exhibits a virtually constant value when the smoothing factor exceeds 0.02. The measured COV values were about 0.012, 0.005, and 0.027 when smoothing factors of 0.01, 0.02, and 0.05 were used, respectively. This means that although the degree of noise reduction is limited, the FNLM algorithm with a high smoothing factor is effective [41]. In addition, we have confirmed that the blurring effect on filtered color LM images is intensified when an FNLM algorithm with a high smoothing factor is applied, as shown in Figure 3. Thus, using an excessively high smoothing factor in the FNLM algorithm can degrade the image characteristics, contrary to the expected effect. As shown in Figure 3b,c, the BRISQUE and NIQE results quantitatively demonstrate the degradation of image characteristics, respectively. As the smoothing factor increases, the NIQE and BRISQUE also continue to increase. The measured BRISQUE values were about 49.44, 57.25, and 60.21 when smoothing factors of 0.01, 0.02, and 0.05 were used, respectively. In addition, the NIQE values were measured as about 4.63, 5.17, and 5.77 when the smoothing factors were set to 0.01, 0.03, and 0.05, respectively. The NIQE and BRISQUE results, which estimate the degree of noise distortions and blur on the image, clearly explain the repercussion of setting excessively high smoothing factors [42,43].

Next, the authors conducted an experiment on the search window and kernel sizes for the FNLM algorithm. As shown in Figure 5a, the increase in the search window and kernel sizes improved the COV, although the effect was less than that of the change in the smoothing factor. Furthermore, the degree of improvement in the COV is more sensitive to the change in the kernel size than that in the search window size. The COV values measured at kernel sizes of 3 × 3 and 11 × 11 are about 0.019 and 0.017 when the search window size was set to 11 × 11, and at window sizes of 11 × 11 and 31 × 31 they are about 0.018 and 0.016 when the kernel size is set to 5 × 5, respectively. However, similar to the smoothing factor experiment, as the search window and kernel sizes increase, the degree of improvement in the COV continues to decrease [44]. As shown in Figure 5b,c, the BRISQUE and NIQE show improved results when a small search window size and a large kernel size are applied, respectively. Especially, the BRISQUE values measured at kernel sizes of 3 × 3 and 11 × 11 are about 51.22 and 50.47 when the search window size is set to 11 × 11, and at window sizes of 11 × 11 and 31 × 31 they are about 50.89 and 53.11 when the kernel size is set to 5 × 5. In addition, the NIQE values measured at kernel sizes of 3 × 3 and 11 × 11 are about 4.66 and 4.53 when the search window size is set to 11 × 11, and at window sizes of 11 × 11 and 31 × 31 they are about 4.59 and 4.68 when the kernel size is set to 5 × 5, respectively. These results indicate that the search window and kernel sizes have a more considerable influence on the blur and noise in the image, respectively [45]. Generally, the search window and kernel sizes of the FNLM algorithm can be set more flexibly when applied to images with regular and repetitive structures; however, in color LM images the structure of specimens is complex and variable. Thus, the search window and kernel sizes should be carefully considered when the FNLM algorithm is applied to color LM images with improved characteristics.

**Figure 6:**
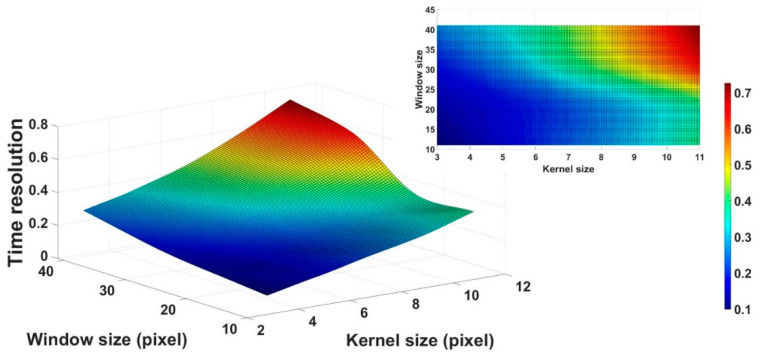
Time resolution results of the FNLM algorithm with various search window and kernel sizes.

In addition, the search window and kernel sizes are closely related not only to image characteristics, but also to the calculation time (i.e., time resolution) [46,47]. Figure 6 shows the time resolution results of various search window and kernel sizes.

According to the results, the time resolution increases as the search window and kernel sizes increase. In particular, the time resolution is approximately 7.39 times the computation times consumed using the lowest (search window size = 11 × 11 and kernel size = 3 × 3) and highest (search window size = 41 × 41 and kernel size = 11 × 11) window and kernel sizes for the FNLM algorithm. Thus, setting high search window and kernel sizes to improve denoising efficiency should be carefully considered, because it can lead to extremely long time resolutions.

**Figure 7:**
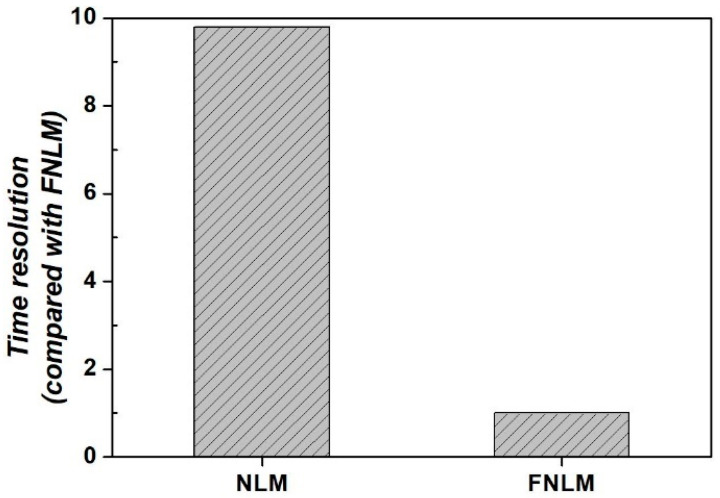
Time resolution results of applying non-local means (NLM) and FNLM algorithms to color LM images.

Moreover, the time resolution of the FNLM algorithm was compared with that of the NLM algorithm to analyze the applicability of the former to color LM images. Figure 7 shows the time resolution results obtained by applying the FNLM and NLM algorithms to color LM images.

As shown in Figure 7, although the results may vary depending on the applied image features, such as matrix size and number of channels, the FNLM algorithm exhibits more improved time resolution results than the NLM algorithm when applied to the same color LM image [48]. In this study, the calculated time resolution of the FNLM algorithm was approximately 9.80 times better than that of the NLM algorithm. The foregoing shows that FNLM algorithm can process color images, which generally have three channels, faster than the NLM algorithm can process single-channel images.

In summary, we derived and analyzed the results with quantitative evaluation for the image characteristics and time resolution of the FNLM algorithm with various experiments. Among these, experiments for image characteristics proved that the FNLM algorithm shows effective effects of noise reduction and reconstructing images in color LM images. In addition, we have shown that the appropriate setting of various parameters is important to increasing the effectiveness of the FNLM algorithm. These results confirmed the feasibility of applying the FNLM algorithm to various medical and scientific fields, such as diagnosis using medical images and optical imaging for nanoscale particle study as well as LM images. However, the color channel separation methods for applying the FNLM algorithm are very classical, and the derived results were not quite satisfying enough to provide dramatic novelty. Furthermore, although the time resolution of the FNLM algorithm showed improved results compared to the conventional NLM algorithm, it showed an insufficient performance to apply to high-frame color LM images for analyzing structural and functional changes of specimens in real time. In the future, we intend to make efforts, such as formula improvement and new framework modeling, to overcome the limitations of the FNLM algorithm mentioned above.

## 5. Conclusions

The authors were led to various conclusions by performing two experiments to analyze the feasibility of the color LM image processing of the FNLM algorithm. First, the high-value parameters of the FNLM algorithm degrade the overall image reconstruction even though the noise problem is improved. Second, excessive parameters settings deteriorated the time resolution of the FNLM algorithm, although the calculation time of the FNLM algorithm was significantly reduced compared to the conventional NLM algorithm. In conclusion, we confirmed that FNLM algorithms with appropriate parameters, considering image characteristics and time resolution, can be usefully applied in color LM images.

## Data Availability

Not applicable.
